# “Precision-CT(O)”: CTO-lesions unraveled by multimodality cardiac imaging

**DOI:** 10.1007/s10554-021-02511-w

**Published:** 2022-01-04

**Authors:** Youssef S. Abdelwahed, Edna Blum, Ulf Landmesser, Gerald S. Werner, David M. Leistner

**Affiliations:** 1 Charité – Universitätsmedizin Berlin, Berlin, Germany; 2 DZHK (German Centre for Cardiovascular Research), Berlin, Germany; 3 German Heart Center Munich, Munich, Germany; 4 Berlin Institute of Health (BIH), Berlin, Germany; 5 Klinikum Darmstadt GmbH, Darmstadt, Germany

## Case

A 67-year-old woman with chronic total occlusions (CTO) located in the proximal RCA and the mid LCx (in-stent-area) presented with recurrent chest pain and dyspnea for recanalization evaluation (Fig. A, B). Stress Echocardiography documented viability in the inferior and lateral myocardial walls.

**Figure:**
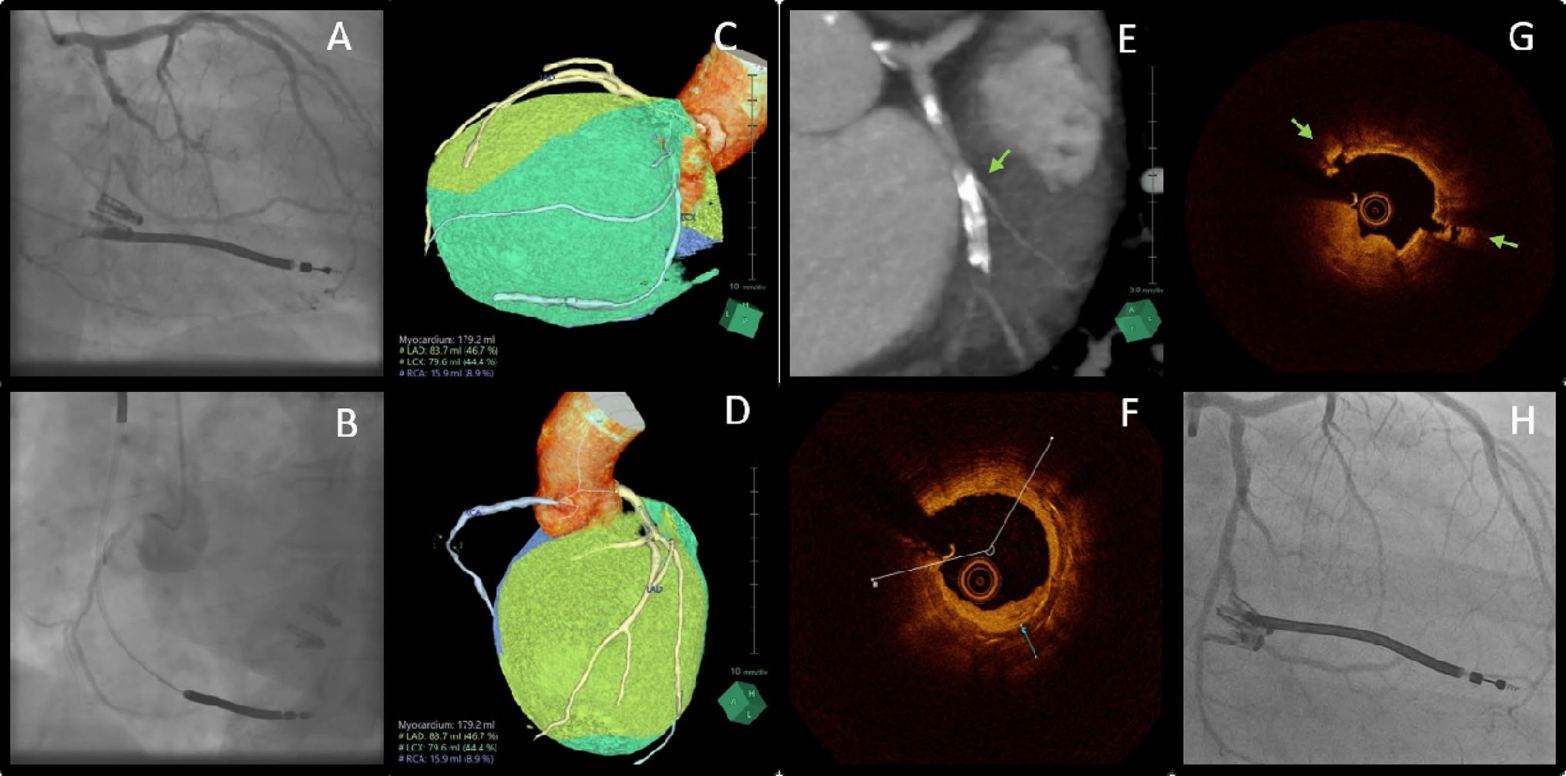
**A** Angiography shows CTO of the mid LCx. **B** Angiography shows the ostial RCA CTO with antegrade collateral filling. **C, D** CT shows a 44.4% supply of the myocardial volume by the LCx and a 8.9% supply by the RCA. **E** CT (Slab-MIP) shows the CTO in the mid LCx with heavy calcification in the proximal part of the CTO segment. (Green Arrow). **F** OCT shows deep calcification with an angle of 250 degrees and thickness of 0.70 mm. **G** OCT shows superficial calcification with cracks (Green arrows) after cutting balloon dilatation and intact deep calcification. **H** Final angiography of the LCx after stents implantation

The revascularization strategy was determined based on Coronary—CT Scan that allowed validation of the supplied areas and the myocardial mass at risk (MMAR) for each artery (Synapse 3D, Fuji Film, Japan) as well as analysis of the particular plaque features. Surprisingly, CT revealed a 44.4% supply of the myocardium by the—heavily calcified—LCx, whereas the RCA supplied only 8.9% of the myocardium (Fig. C–E). Consecutively, recanalization of the LCx was pursued; while the RCA should be left to OMT.

Crossing of the CTO-segment was successfully performed following an antegrade wire escalation technique under support of Finecross Microcatheter (Terumo, Japan) using the Abbott-Infiltrac wire (HI-TORQUE INFILTRAC™). After cap dilatation with compliant followed by non-compliant balloons, Optical-Coherence Tomography (OCT) within the CTO-segment was performed, revealing superficial and—most importantly—deep calcification (thickness: 0.7 mm; length: 9 mm; Angle: 250 degrees) (Fig. F).

Subsequently a differentiated OCT-based debulking strategy was performed including cutting balloon angioplasty (WOLVERINE™, Boston Scientific, USA) against superficial calcium (Fig. G) followed by intracoronary lithoplasty (S-IVL; Shockwave Medical, Inc, USA) to treat the deep calcified vessel layers.

Finally—following the MLD—MAX (Morphology, Length, Diameter-Medial dissection, Apposition, Xpansion)—precision-PCI-algorithm—successful PCI of the LCx (SMT Cruz Supraflex 3.0 × 24 mm) with excellent result (Figu. H) was achieved.

This case introduces the concept of “Precision-CT(O)” as a novel strategy to tailor the revascularization strategy in complex CAD including CTO-lesion(s). By integrating pre-procedural CT-assessment and peri-procedural OCT-PCI-guidance, “Precision-CT(O)” may be an option to individualize the CTO recanalization strategy and to improve long-term results after CTO-PCI.

